# Obesity: A Complex Process Requiring Reframing

**DOI:** 10.1177/2150132720979142

**Published:** 2020-12-10

**Authors:** Jay Anil Patel, Ashni Asit Badiani

**Affiliations:** 1 London School of Hygiene and Tropical Medicine, London, UK; 2 School of Medicine, University of Southampton, Southampton, UK; 3 School of Medicine, University of Liverpool, Liverpool, Merseyside, UK; 4 University College London, London, UK

**Keywords:** global health, health promotion, health outcomes, lifestyle change, long term care, obesity, patient-centeredness, physical activity, practice management, prevention

Dear Editor,

Thank you for publishing the article, “Childhood Obesity: An Evidence-Based Approach to Family Centered Advice and Support,” in your journal, which was a particularly engaging piece.^1^ The article discussed how childhood obesity can be combatted through patient-centered behavioral approaches and emphasized the role of primary care physicians in tackling the obesity epidemic. However, the article also highlighted how biases amongst physicians can lead to stigmatizing beliefs^1,2^ which blame individuals for exercising insufficiently and eating excessively. This attitude of victimization is unhelpful. First, stigmatizing people with obesity can reduce the effectiveness of weight loss and obesity intervention efforts.^1,2^ Second, it can prevent individuals accessing healthcare and lead to psychological distress.^1,2^ Third, this attitude ignores the upstream determinants of obesity, making it more challenging to tackle them.

Although Kaufman et al.^1^ state that many physicians understand that tackling the obesity epidemic requires both public health and social interventions, they also recognize that knowledge regarding obesity remains a major issue amongst primary care physicians. Therefore, this article will attempt to provide a systems-based overview of the upstream determinants of the obesity epidemic, attempting to redefine the condition away from its focus on individuals and towards a structured approach which considers both its upstream and downstream determinants.

## Built Environment & Land Use

On a macroscopic level, the environment where people live can affect their risk of developing obesity. People living in areas with abundant greenspace availability have lower rates of obesity, likely because this greenspace facilitates increased physical activity.^3^ Also, the location where people reside affects their likelihood of having obesity. Living in areas where land is well distributed between residential, commercial and industrial property encourages the use of active forms of transport (such as walking or cycling), which increase physical activity and protect against obesity.^4,5^

## Incomes, Poverty, and Socioeconomic Circumstances

The influential Marmot Review showed that deprivation is associated with an increased risk of obesity across the income distribution spectrum.^6^ Two main theories are used to explain this association. The increased likelihood of health-risk behaviors, such as physical inactivity or excessive alcohol intake, amongst people living in poverty and the material circumstances of poverty itself, such as a lack of access to nutritious foods, likely explain how poverty contributes to the development of obesity. Therefore, inequalities remain a key determinant of obesity, and tackling this epidemic requires policymakers to address these underlying issues.

## Obesity: An Economic Perspective

Industrialization, mass-scale farming and new technologies have improved agricultural yields, reducing the cost of food production and transport, as well as increasing the supply of food.^7^ With increased food availability, prices haves dramatically fallen since The Industrial Revolution.^8^ Food prices affect consumption, hence why taxes on unhealthy foods can be effective.^9^ Therefore, falling prices have increased the consumption of food and the likelihood of excessive energy intake, increasing the risk of people developing obesity.

Since the Industrial Revolution, many occupations have become less physically demanding due to new technologies, machinery and changing labor patterns, away from manual labor and towards office-based roles.^8^ Therefore, physical activity has fallen in the workplace, with people no longer working (and earning money) in occupations where they must exercise. This fall in workplace physical activity has not been replaced by increased leisure-based physical activity, which requires people’s time and money. Overall, the rise in the monetary and time cost of exercise have unsurprisingly led to falling physical activity and energy expenditure over time, increasing the likelihood of people developing obesity.

## The Commercial Determinants of the Obesity Epidemic

Sir David King stated that the obesity epidemic “reflects a failure of the free market,” a commercial success for the food industry but a health catastrophe. In the relentless drive for profits, manufacturers of unhealthy foods (such as sugar sweetened beverages, junk, and fast foods) have disregarded the population’s health. For example, marketing, which commonly promotes unhealthy foods, has shaped individual food preferences, driving junk and unhealthy food consumption, fueling the obesity epidemic.^10^

## Sociological Determinants of Obesity: Social Networks and Culture

Obesity is known to spread through social networks over time, particularly via siblings and same-sex friendships.^4,11^ This may be mediated by social contagion, where people’s social networks influence their obesity-related behaviours^4^—for example, non-obese people may mirror the unhealthy behaviors and bodyweight ideals of friends who are overweight, promoting the development of obesity.^12^ On the other hand, feeling a sense of belonging to a group (e.g., in community organizations and volunteering) may protect against the development of obesity.^12^ Also, considering how support from friends and family can lead to a greater number of healthy behaviors, a lack of social support can be a barrier to weight loss, hindering efforts to tackle obesity.^12^

Culture remains an important driver of the obesity epidemic, and cannot be ignored. It shapes attitudes towards food, defining what items are considered healthy and unhealthy and influencing individuals’ dietary choices. Food is tied to one’s identity, and consuming traditional foods can help to maintain cultural identity within immigrant populations^13^—depending upon the healthfulness of these foods, they can either increase or decrease obesity risk.^13^ Alternatively, attempts to assimilate into Western society can lead to others abandoning traditional diets and adopting energy-dense Westernized diets, increasing their risk of obesity.^13^ Also, attitudes towards body image are critical, particularly amongst ethnic minorities, who may idealize being overweight, viewing it as a sign of health and wealth, such as African-Americans.^13^ Considering cultural attitudes to health and idealized body size are important drivers of obesity amongst minority groups, they cannot be ignored.

## Evolution: A Genome Unsuited to a World of Excess

The human genome is designed for a hunter-gatherer lifestyle, with “thrifty genes” adapted to conserve energy to stave off the negative effects of food shortages.^7^ For example, humans are genetically predisposed towards over-eating rather than under-eating, with the body better at signaling hunger rather than feeling satiated. Evolution has created a human body predisposed to overeating and weight gain, which would have conserved energy in preparation for food shortages. Yet, in today’s society, with an ever-present availability of cheap, energy-dense food, it has helped to fuel the obesity epidemic.

## Conclusion

Obesity is not a linear problem. It can no longer be viewed with negative attitudes and victimization of people with obesity must end. Policymakers must view the obesity epidemic as a multidimensional problem which requires a systems-based approach alongside lifestyle and dietary interventions. Tackling the epidemic is as much about individual choice as optimizing the environment, addressing socioeconomic barriers to health and recognizing the cultural drivers of obesity.
